# Delay in the initiation of adjuvant chemotherapy in patients with breast cancer with mastectomy with or without immediate breast reconstruction

**DOI:** 10.1093/bjsopen/zrac096

**Published:** 2022-08-11

**Authors:** Tuomas Huttunen, Marjut Leidenius, Tiina Jahkola, Johanna Mattson, Sinikka Suominen, Tuomo Meretoja

**Affiliations:** Department of Breast Surgery, Comprehensive Cancer Center, University of Helsinki and Helsinki University Hospital, Helsinki, Finland; Department of Breast Surgery, Comprehensive Cancer Center, University of Helsinki and Helsinki University Hospital, Helsinki, Finland; Department of Plastic Surgery, University of Helsinki and Helsinki University Hospital, Helsinki, Finland; Comprehensive Cancer Center, University of Helsinki and Helsinki University Hospital, Helsinki, Finland; Department of Plastic Surgery, University of Helsinki and Helsinki University Hospital, Helsinki, Finland; Department of Breast Surgery, Comprehensive Cancer Center, University of Helsinki and Helsinki University Hospital, Helsinki, Finland

## Abstract

**Background:**

Patients with breast cancer undergoing mastectomy should be offered the option of immediate breast reconstruction (IBR). The aim of this retrospective study was to assess whether there is a delay in the initiation of adjuvant chemotherapy in patients undergoing mastectomy with or without IBR.

**Method:**

The study included patients aged 70 years or younger with clinically node-negative breast cancer who underwent unilateral mastectomy with IBR (IBR group) or mastectomy alone (no-IBR group) followed by adjuvant chemotherapy at the Helsinki University Hospital between January 2012 to July 2018.

**Results:**

A total of 645 patients were included; 186 in the IBR group and 459 in the no-IBR group. Sixty-six (35.5 per cent) patients in the IBR group and 102 (22.2 per cent) patients in the no-IBR group received their first chemotherapy cycle later than 6 weeks after surgery (*P* < 0.001). The respective numbers for later than 8 weeks were 17 (9.1 per cent) and 14 (3.1 per cent) (*P* = 0.001). Among all 645 patients, postoperative complications were a significant risk factor for a delay in the initiation of chemotherapy. Sixty-seven (39.9 per cent) patients with and 101 (21.2 per cent) patients without complications had a delay in chemotherapy (*P* < 0.001). The delay in chemotherapy was due to complications in 39 (59.1 per cent) in the IBR group and in 28 (27.5 per cent) in the no-IBR group (*P* < 0.001).

**Conclusion:**

Patients undergoing mastectomy alone were more likely to receive adjuvant chemotherapy within 6 weeks after surgery compared with the IBR patients. IBR significantly increased the risk of postoperative complications in comparison with mastectomy alone. The complications, in turn, were a significant risk factor for delay in adjuvant chemotherapy.

## Teaser

The aim of this retrospective study was to see whether immediate breast reconstruction (IBR) is linked to a delay in initiation of adjuvant chemotherapy. The study shows that patients undergoing mastectomy alone were more likely to receive adjuvant chemotherapy within the recommended 6 weeks after surgery when compared with IBR patients. IBR significantly increased the risk of postoperative complications and the complications, in turn, were a significant risk factor for delay in adjuvant chemotherapy.

## Introduction

Patients with breast cancer undergoing mastectomy should be offered the option of immediate breast reconstruction (IBR). IBR has proven to give psychological benefits to patients and increase quality of life in comparison with mastectomy alone^1,2^; however, reconstructive surgery includes a greater risk of complications in comparison with mastectomy alone^3^. Typical postoperative complications include haematoma, infection, mastectomy skin flap necrosis, implant loss, and vascular problems with the flap reconstruction^4,5^.

Complications may delay the initiation of adjuvant chemotherapy. There is not a clear consensus of the timing of adjuvant chemotherapy^6^. The European Society for Medical Oncology (ESMO) recommends that adjuvant systemic treatment should start within 6 weeks after surgery^7^. A longer delay to chemotherapy can worsen prognosis, especially in patients with higher tumour stage or biologically aggressive cancer^5,8–11^. Many previous studies have reported an increased complication rate after IBR when compared with mastectomy alone, but these results have been controversial regarding whether the increased complication rate has an impact on the timing of adjuvant chemotherapy^3,12–22^.

This study aims to clarify whether there is a delay in adjuvant chemotherapy in patients who undergo mastectomy and IBR.

## Methods

The patients in this retrospective study were identified from Helsinki University Hospital (HUH) electronic patient database; therefore, no approval of the Ethics Committee of HUH was needed. The institutional research permission was granted by HUH Comprehensive Cancer Center.

### Patients

The study included patients with clinically node-negative breast cancer, who were 70 years old or younger and underwent unilateral mastectomy with IBR (the IBR group) or mastectomy alone (the no-IBR group) followed by adjuvant chemotherapy at HUH from January 2012 to July 2018. The following patients were excluded: those who received neoadjuvant chemotherapy or underwent mastectomy after failed breast conservation; patients who underwent axillary lymph node dissection (ALND) as a re-operation before adjuvant chemotherapy; those with a recurrent cancer; patients who did not undergo chemotherapy after the operation; and those with bilateral cancers as well as patients undergoing bilateral mastectomies.

The collected data included the age of the patient, the date of operation, the date of the first chemotherapy cycle, tumour characteristics, including oestrogen receptor (ER), progesterone receptor (PR), proliferation index (Ki-67), human epidermal growth factor receptor 2 (HER2), tumour size, histological tumour type, nodal status, type of axillary surgery, the method of breast reconstruction, and possible symmetrizing operation to the contralateral breast. Furthermore, patients’ BMI, diabetes status, and smoking status were collected. Furthermore, data regarding postoperative complications were collected. Complications included infection, postoperative haematoma, mastectomy skin flap necrosis, IBR flap necrosis, and IBR loss. Furthermore, information regarding re-operations due to complications was collected.

### Surgery

All patients were operated on by experienced breast surgeons and plastic surgeons. All mastectomies were performed with an aim to remove the breast tissue as completely as possible at the level of the superficial fascia with an effort not to endanger the vitality of the skin envelope. The IBR methods included pedicled latissimus dorsi (LD) flaps, LD flaps combined with implants or with free-fat transplant, microvascular abdominal flaps (transverse rectus myocutaneous (TRAM) flap, deep inferior epigastric artery perforator (DIEP) flap and superficial inferior epigastric artery (SIEA) flap), microvascular thigh flaps (transverse myocutaneous gracilis (TMG) flap, also called transverse upper gracilis flap) or with one- or two-staged implant reconstruction. At the study unit, IBR was not recommended in patients who were likely to receive postoperative radiation therapy (those with clinical stage T3 or 4) or clinically positive axilla. Furthermore, co-morbidities and other risk factors as well as patients’ preferences were taken into consideration when the option of IBR was discussed with the patient. All patients underwent sentinel node biopsy (SNB), either alone or followed by ALND during the same operation due to a tumour-positive sentinel node in the intraoperative frozen section diagnosis.

### Histopathology

Histopathological analyses from surgical specimens were performed by experienced breast pathologists as described in a previous study^23^.

### Statistical methods

Statistical analyses were performed with SPSS^®^ version 26 (IBM, Armonk, New York, USA). Frequency tables were analysed with the chi-squared test and continuous variables were compared with the Mann–Whitney *U* test.

## Results

### Patients and surgery

A total of 645 patients were included; 186 in the IBR group and 459 in the no-IBR group.

Of the 186 patients who underwent IBR, 161 had skin-sparing mastectomy (SSM) and 25 had nipple–areola complex-sparing mastectomy (NSM). Of these, 71 (38.2 per cent) underwent a free-flap reconstruction (DIEP, TRAM, SIEA, or TMG), 74 (39.8 per cent) underwent an LD flap reconstruction (also including those combined with an implant or free-fat transplantation), and 41 (22.0 per cent) underwent an implant/expander prosthesis reconstruction. Two-stage expander reconstruction was performed in 35 (82.9 per cent) of the patients with implant reconstruction. Eighteen (72.0 per cent) of the NSM patients underwent an implant reconstruction.

All patients, both in the IBR group and in the no-IBR group, underwent SNB. In the IBR group, 93 (50.0 per cent) patients and in the no-IBR group 275 (59.9 per cent) patients underwent additional ALND (*P* = 0.021).

The patients in the IBR group were younger compared with the no-IBR group. The patients in the IBR group also had a lower median BMI, and fewer of them had diabetes, or were smokers. The pT and pN categories were more advanced in the no-IBR group patients. There were no significant differences in the tumour grade or ER, PR, and HER2 status between the IBR and the no-IBR group (*Table 1*).

**Table 1:** Patient and tumour characteristics for the patients with immediate breast reconstruction and for the patients with mastectomy alone

	IBR	No-IBR	*P*
	*n* = 186	*n* = 459	
**Patient age (years)**			<0.001
**Median (range)**	48 (23–70)	54 (24–70)	
**50 years old or younger**	120 (64.5)	184 (40.1)	<0.001
**More than 50 years old**	66 (35.5)	275 (59.9)	
**Diabetes**			0.314
Yes	4 (2.2)	17 (3.7)	
No	182 (97.8)	442 (96.3)	
**Smoking**			<0.001
Yes	15 (8.1)	86 (18.7)	
No	171 (91.9)	373 (81.3)	
**BMI**			0.017
**Median (range)**	23.6 (17.2–40.9)	24.2 (15.6–42.6)	
**pT category**			<0.001
Tmic	1 (0.5)	0 (0)	
T1 (a–c)	106 (57.0)	156 (34.0)	
T2	63 (33.9)	237 (51.6)	
T3	15 (8.1)	63 (13.7)	
T4	1 (0.5)	3 (0.7)	
**pN category**			<0.001
N0	85 (45.7)	144 (31.4)	
N1 and N1mi	85 (45.7)	219 (47.8)	
N2–3	16 (8.6)	96 (20.9)	
**Histological type**			<0.001
Ductal	135 (72.6)	245 (53.4)	
Lobular	32 (17.2)	145 (31.6)	
Other invasive	19 (10.2)	69 (15.0)	
**Tumour grade**			0.606
1	22 (11.8)	54 (11.7)	
2	78 (41.9)	174 (37.9)	
3	86 (46.2)	231 (50.3)	
**ER**			0.973
Positive	155 (83.3)	383 (83.2)	
Negative	31 (16.7)	77 16.8)	
**PR**			0.144
Positive	128 (68.8)	288 (62.7)	
Negative	58 (31.2)	171 (37.3)	
**HER2**			0.261
Positive	46 (24.7)	95 (20.7)	
Negative	140 (75.3)	364 (79.3)	
**Biological subtype**			0.452
ER/PR^+^, HER2^−^	125 (67.2)	311 (67.8)	
HER2^+^	45 (24.2)	96 (20.9)	
ER^−^PR^−^HER2^−^	16 (8.6)	52 (11.3)	
**Ki-67 (%)**			0.549
0–15	52 (28.0)	111 (24.2)	
16–30	55 (29.6)	135 (29.4)	
>30	79 (42.5)	213 (46.4)	
**Lymphovascular invasion**			0.033
Yes	39 (21.0)	134 (29.2)	
No	147 (79.0)	325 (70.8)	
**Axillary surgery**			0.021
SNB + ALND	93 (50.0)	275 (59.9)	
SNB only	93 (50.0)	184 (40.1)	
**Symmetry procedure**			<0.001
Yes	24 (12.9)	16 (3.5)	
No	162 (87.1)	443 (96.5)	
**LMWH prophylaxis**			<0.001
Yes	150 (80.6)	33 (7.2)	
No	36 (19.4)	426 (92.8)	

Values are *n* (%) unless otherwise indicated. ALND, axillary lymph node dissection; SNB, sentinel node biopsy; ER, oestrogen receptor; PR, progesterone receptor; HER2, human epidermal growth factor receptor; Ki-67, proliferation marker; LMWH, low molecular weight heparin.

### Complications

Seventy-one (38.2 per cent) patients in the IBR group and 97 (21.1 per cent) patients in the no-IBR group had at least one complication (*P* < 0.001). Fifty-nine (36.6 per cent) patients who underwent SSM and 12 (48.0 per cent) patients who underwent NSM had a complication (*P* = 0.277). Twenty-seven (38.0 per cent) patients with a microvascular reconstruction, 31 (41.9 per cent) patients with an LD reconstruction, and 13 (31.7 per cent) patients with an implant reconstruction had a complication (*P* = 0.560). Five out of 41 (12.2 per cent) patients with implant reconstruction had an implant loss. There were no autologous flap losses (0 of 145; *P* < 0.001). Two patients, one with an LD reconstruction and another with a microvascular reconstruction, had a partial flap necrosis.

**Fig. 1:**
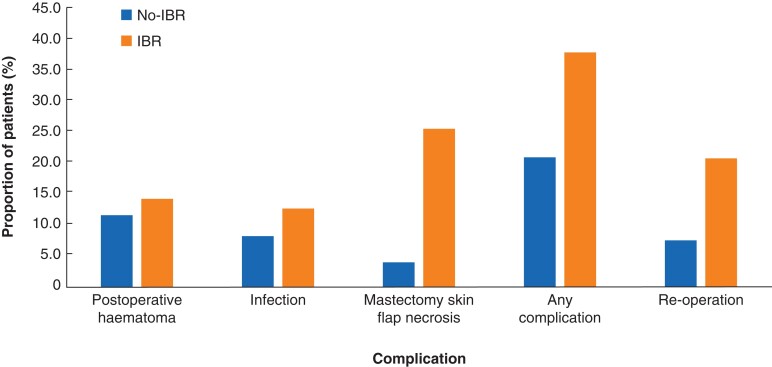
Proportion of patients with complications IBR, immediate breast reconstruction.

All complications were related to wound healing. There were no general postoperative complications such as pneumonia or embolism in either of the groups. The most common complication was mastectomy skin flap necrosis that occurred in 48 (25.8 per cent) IBR patients but only in 18 (3.9 per cent) no-IBR group patients (*P* < 0.001). The proportions of patients with complications are shown in *Fig. 1*.

The number of re-operations due to complications was also significantly higher in the IBR group than in the no-IBR group, 39 (21.0 per cent) and 35 (7.6 per cent) respectively (*P* < 0.001). Most re-operations were wound revisions and evacuation of haematomas.

### Initiation of chemotherapy

Sixty-six (35.4 per cent) IBR patients and 102 (22.2 per cent) no-IBR group patients received their first chemotherapy cycle later than 6 weeks after their operation (*P* < 0.001) (*Table 2*). Chemotherapy was delayed in 33 (46.5 per cent) patients with a microvascular reconstruction, in 26 (35.1 per cent) patients with an LD reconstruction, and in seven (17.1 per cent) patients with an implant reconstruction (*P* = 0.007).

**Table 2:** Initiation times to first adjuvant chemotherapy cycle after surgery and reasons for the delay

Time to first chemotherapy	IBR	No-IBR	*P*
**Days, median (range)**	40 (26–89)	37 (20–120)	0.004
**Before or after 6 weeks**			<0.001
<6 weeks	120 (64.5)	357 (77.8)	
>6 weeks	66 (35.5)	102 (22.2)	
**Reason for initiation >6 weeks**			0.002
Wound complication	39 (59.1)	28 (27.5)	
Other medical reason*	9 (13.6)	32 (31.4)	
Hospital resource related†	9 (13.6)	21 (20.6)	
Patient preference	3 (4.5)	7 (6.7)	
Unknown	6 (9.1)	14 (13.7)	

Values are *n* (%) unless otherwise indicated.

* Includes such reasons as infections not related to surgery, cardiological examinations, and further examinations due to findings in staging CT.

† The most common hospital resource-related reason was a delay in histopathology, especially in HER2 amplification testing by *in situ* hybridization.

IBR, immediate breast reconstruction; HER2, human epidermal growth factor receptor.

The delay in chemotherapy was due to wound complications in 39 (59.1 per cent) IBR group patients and in 28 (27.5 per cent) no-IBR group patients, (*P* < 0.001). Twenty-seven (40.9 per cent) IBR group patients and 74 (72.5 per cent) no-IBR group patients had a delay due other reason. The median time to initiation of chemotherapy was 37 (range 20–120) days in the no-IBR group and 40 (range 26–89) days in the IBR group (*P* = 0.004).

Seventeen (9.1 per cent) IBR group patients received their first chemotherapy cycle later than 8 weeks after surgery. In 11 of them, the reason for the delay was a wound complication. Fourteen (3.1 per cent) no-IBR group patients received their first chemotherapy cycle after 8 weeks. In six patients the reason for the delay was a wound complication.

Delay in the initiation of chemotherapy beyond 6 weeks was observed in 168 (26.0 per cent) patients in total (*Table 3*). Postoperative complications were a significant risk factor for delay. Sixty-seven (39.9 per cent) patients with and 101 (21.2 per cent) patients without a complication had a delay (*P* < 0.001). Skin envelope necrosis and infections both were significant risk factors for a delay. Thirty-eight (57.6 per cent) patients with skin necrosis had a delay, whereas 130 (22.5 per cent) of the patients without skin necrosis had a delay (*P* < 0.001). The respective figures for infections were 37 (59.7 per cent) and 131 (22.5 per cent; *P* < 0.001).

**Table 3:** Risk factors for the delay of adjuvant chemotherapy

	Initiation > 6weeks (*n* = 168)	No delay (*n* = 477)	*P*
**Patient group**			<0.001
IBR	66 (35.5)	120 (64.5)	
no IBR	102 (22.2)	357 (77.8)	
**Patient age (years)**			0.004
*≤*50	63 (20.7)	241 (79.3)	
>50	105 (30.8)	236 (69.2)	
**Diabetes**			0.074
Yes	9 (42.9)	12 (57.1)	
No	159 (25.5)	465 (74.5)	
**Smoking**			0.247
Yes	31 (30.7)	70 (69.3)	
No	137 (25.2)	407 (74.8)	
**BMI**			0.964
>30	17 (25.8)	49 (74.2)	
≤30	140 (25.5)	409 (74.5)	
**Mastectomy skin flap necrosis**			<0.001
Yes	38 (57.6)	28 (42.4)	
No	130 (22.5)	449 (77.5)	
**Haematoma**			0.556
Yes	23 (28.8)	57 (71.3)	
No	145 (25.7)	420 (74.3)	
**Infection**			<0.001
Yes	37 (59.7)	25 (40.3)	
No	131 (22.5)	452 (77.5)	
**Any complication**			<0.001
Yes	67 (39.9)	101 (60.1)	
No	101 (21.2)	376 (78.8)	
**Re-operation due to a complication**			<0.001
Yes	34 (45.9)	40 (54.1)	
No	134 (23.5)	437 (76.5)	
**Axillary surgery**			0.109
SNB + ALND	87 (23.6)	281 (76.4)	
SNB only	81 (29.2)	196 (70.8)	
**Symmetry procedure**			0.005
Yes	18 (45.0)	22 (55.0)	
No	150 (24.8)	455 (75.2)	
**LMWH prophylaxis**			<0.001
Yes	68 (37.2)	115 (62.8)	
No	100 (21.6)	362 (78.4)	

Values are *n* (%). IBR, immediate breast reconstruction; ALND, axillary lymph node dissection; SNB, sentinel node biopsy; LMWH, low molecular weight heparin.

With regard to patient characteristics, only older patient age was a statistically significant risk factor for the delay. One hundred and five (30.8 per cent) of the patients who were more than 50 years old had a delay, whereas 60 (20.7 per cent) of the patients who were 50 years old or younger had a delay (*P* = 0.004). Smoking, diabetes, BMI more than 30, and ALND were slightly more common in patients who had a delay, but these findings were not statistically significant.

Surgery to the contralateral breast was associated with an increased risk of complications. Nineteen (47.5 per cent) of the 40 patients with surgery to the contralateral breast and 149 (24.6 per cent) patients without it had at least one complication (*P* = 0.001). The complication was on the contralateral side in three (15.8 per cent) cases, on both sides in three (15.8 per cent) cases, and on the ipsilateral side in 13 (68.4 per cent) cases.

## Discussion

This study shows that patients who underwent mastectomy alone were more likely to receive adjuvant chemotherapy within the recommended 6 weeks from surgery when compared with the IBR patients. On the other hand, at least 90 per cent of the patients received their first chemotherapy cycle within 8 weeks, even in the IBR group. There is no clear consensus of the optimal timing of adjuvant chemotherapy for patients with breast cancer and the data regarding this are controversial^6^. There are no randomized clinical trials addressing the timing to start adjuvant chemotherapy for obvious ethical reasons. A systematic review and meta-analysis by Raphael *et al.* showed that a 4-week increase in time to adjuvant chemotherapy was associated with a significant increase in the risk of death^11^. On the other hand, a retrospective review by Lorisch *et al.*^24^ in early-stage breast cancer suggests that adjuvant chemotherapy is equally effective up to 12 weeks after surgery. ESMO recommends that adjuvant systemic treatment should start within 6 weeks after surgery^7^. This is also the recommendation of the Finnish Institute for Health and Welfare^25^. It is possible that the delay in the present study is not clinically significant for many patients; however, for patients with higher tumour stages or biologically aggressive tumours, even a short delay may affect the outcome^8^. Therefore, any unnecessary delay in initiation of chemotherapy should be avoided.

Most previous studies have shown controversial findings about the influence of IBR on the timing of adjuvant chemotherapy. A systematic review from 2015^20^ concluded that IBR does not delay adjuvant chemotherapy in a clinically relevant way, although the studies included had controversial findings. On the other hand, the findings of the present study are in close agreement with a recent Dutch nationwide population-based study^19^. Unlike in the present study, 91 per cent of the reconstructions were implant based^19^. Nevertheless, Kontos *et al*. found a significant delay in the commencement of adjuvant treatment after mastectomy and free-flap IBR in comparison with patients with mastectomy alone due to reconstruction-related surgical complications, although their study population was relatively small^22^. In this study, delays were more frequent among patients who underwent a microvascular reconstruction when compared with other reconstruction methods. Microvascular surgery takes a longer time and technical problems can occur more often, which further increase the risk for infections and other complications.

In the present study, IBR significantly increased the risk of postoperative complications in comparison with mastectomy alone. The complications, in turn, were a significant risk factor for delay in adjuvant chemotherapy in all patients. Eck *et al*. found that when complications occur, adjuvant therapy is significantly delayed, and that IBR almost doubles the complication risk^21^. Mortenson *et al.* showed an increased incidence of wound complications in patients undergoing IBR^12^; however, neither of the studies^12,21^ reported a delay in initiation of postoperative chemotherapy in IBR patients, but the numbers of patients who received adjuvant chemotherapy after IBR were significantly lower (47 and 83) than in the present study^12,21^.

Older patient age, smoking, diabetes, and obesity are well known risk factors for postoperative complications^5,26,27^. In the present study, older patient age was a significant risk factor for both complications and chemotherapy delay, although the IBR group patients were younger than those with mastectomy only. Surprisingly, there was no statistically significant difference in the smoking status, diabetes, or BMI between patients who had a delay in adjuvant chemotherapy and those who did not. Most likely this was because smokers, obese patients, and those who had diabetes received IBR less often. It is noteworthy, that complications were more frequent in the IBR group, although IBR patients had these risk factors less frequently.

Mastectomy skin flap necrosis was a frequent complication in this study. It was relatively high especially in the IBR group (25.8 per cent). It also led to a high re-operation rate in the IBR group. In a review by Robertson *et al.* the rate for mastectomy skin flap necrosis for mastectomy with IBR ranged from 7–30 per cent^28^. At the study centre, all mastectomies are performed at the level of the superficial fascia without any threshold thickness for the skin flaps, emphasizing the removal of breast tissue as completely as possible but without endangering the vitality of the skin envelope. The odds of getting postoperative skin necrosis seems to be more than six-times higher in patients with skin flap thickness less than 5 mm^29^. On the other hand, there is a high prevalence of residual glandular tissue and even residual disease with skin flaps thicker than 5 mm^30^. According to a recent study with breast MRI, residual breast tissue is left behind in all mastectomy types, but most frequently after NSM^31^.

The present strategy in the study centre to avoid skin necrosis is to preserve the subdermal vascular plexus of the skin envelope intact from surgical and thermal injury. It is also crucial to stay within ‘the breast footprint’, and not divide perforating vessels of the surrounding skin when performing mastectomy. When dermal ischaemia is suspected, an intraoperative indocyanine green (ICG) fluorography device has proven to be useful when excising the damaged skin during the primary operation^32^.

To avoid any delays in adjuvant treatments, active treatment of complications is crucial regardless of whether the patient has received IBR. In the present study, some of the patients also had a delay due to other medical reasons. This occurred especially in the no-IBR group where the patients were older. It is important to consider and actively treat other medical conditions to avoid delays in adjuvant treatment. Some of the patients also had a delay due to hospital resource-related reasons that should not occur.

The delay in adjuvant chemotherapy leads to an unfavourable prognosis, especially in patients with higher tumour stages or biologically aggressive tumours^8–11,33^. On the other hand, neoadjuvant chemotherapy is currently preferred in these patients.

The limitation of this study was the retrospective, non-randomized setting. In addition, a selection bias was clear, although patients older than 70 years and those with clinically positive axilla as well as those with bilateral mastectomies were excluded; however, the selection bias favoured the IBR group, and thus did not influence the conclusions. The influence of the complications or the timing of adjuvant chemotherapy on prognosis has not been evaluated in this study and it is not clear whether the delay makes a clinical difference to patients.

Patients who undergo mastectomy alone are more likely to receive adjuvant chemotherapy within the recommended 6 weeks from surgery when compared with IBR patients. IBR significantly increased the risk of postoperative complications in comparison with mastectomy alone and the complications, in turn, were a significant risk factor for delay in adjuvant chemotherapy.

## Data Availability

Data are not available to other researchers. The research was not pre-registered with an analysis plan in an independent institutional registry.
